# Prevention of end‐stage renal disease with percutaneous endoscopic gastrojejunostomy tubes in a patient with recurrent episodes of binge eating and purging

**DOI:** 10.1002/pcn5.63

**Published:** 2022-11-30

**Authors:** Kazuhito Takahashi, Takaaki Shimizu, Daimei Sasayama, Shinsuke Washizuka

**Affiliations:** ^1^ Department of Psychiatry Shinshu University School of Medicine Matsumoto Nagano Japan; ^2^ Department of Psychiatry Ritsuzankai Iida Hospital Iida Nagano Japan; ^3^ Department of Psychiatry Nagano Red Cross Hospital Nagano Japan; ^4^ Department of Child and Adolescent Developmental Psychiatry Shinshu University School of Medicine Matsumoto Nagano Japan

**Keywords:** binge‐eating/purging subtype of anorexia nervosa, bulimia nervosa, end‐stage renal disease, percutaneous endoscopic gastrojejunostomy

## Abstract

**Background:**

Bulimia nervosa (BN) and the binge‐eating/purging subtype of anorexia nervosa (b/p AN) are characterized by binge eating and unsafe compensatory behaviors, such as laxatives or diuretic abuse, self‐induced vomiting, and excessive exercise. BN often causes miscellaneous physical complications that can lead to death. However, there have been very few prior reports on the physical complications of chronic BN.

**Case Presentation:**

We report a case of chronic BN of over 10 years. Her compensatory behaviors caused dehydration more easily than before. Repeated dehydration may result in end‐stage renal disease (ESRD). Therefore, we had to prevent dehydration by placing a percutaneous endoscopic gastrojejunostomy (PEG‐J) tube. After PEG‐J placement, dehydration did not reoccur, thereby protecting her renal involvement from progressing to ESRD.

**Conclusion:**

Chronic BN exposure may cause repeated dehydration with time. Therefore, the PEG‐J tube would be effective in preventing dehydration, which leads to ESRD.

## BACKGROUND

Bulimia nervosa (BN) and the binge‐eating/purging subtype of anorexia nervosa (b/p AN) are characterized by binge eating and unsafe compensatory behaviors, such as self‐induced vomiting, laxative abuse, and excessive exercise. These characteristics cause several physical complications, for example, hypokalemia, renal involvement, liver dysfunction, and gastrointestinal symptoms.^1^ Although the complications are usually transient, if they are not treated appropriately, they sometimes result in death. For example, self‐induced vomiting and laxative abuse can cause hypokalemia, which results in prolonged QTc interval, leading to severe ventricular tachycardia, known as torsades de pointes.^1^ A previous report described the mechanisms of progressing end‐stage renal disease (ESRD) in a chronic BN patient who finally died of septicemia.^2^ In terms of prognosis, a 10‐year follow‐up study showed that 52% of BN patients made a full recovery and the full symptoms of BN continued in 9% of the patients.^3^ However, there are few reports of BN patients who developed ESRD, which requires maintenance hemodialysis.^4^ Moreover, there are no reports on ways to prevent the development of ESRD in patients with chronic BN. Despite numerous reports of acute renal failure in patients with eating disorders, the development of ESRD due to chronic BN may be rare.

Percutaneous endoscopic gastrojejunostomy (PEG‐J) is a common procedure that allows jejunal feeding in patients with restricted oral intake who require long‐term nutritional support. The percutaneous endoscopic gastrostomy (PEG) tube is placed directly into the stomach through the abdominal wall, and the PEG‐J tube is an extension tube inserted into the jejunum via the existing PEG tube.^5^

We report the case of a woman with chronic BN. Her compensatory behaviors, such as laxative abuse and self‐induced vomiting, caused repeated dehydration. As her renal function was about to deteriorate to ESRD, protecting her from dehydration was crucial. To prevent dehydration, a percutaneous endoscopic gastrojejunostomy (PEG‐J) tube was placed. The PEG‐J tube allowed her to consume stable calories and sufficient fluid volume. Consequently, she was able to avoid progressing to ESRD.

## CASE PRESENTATION

A 36‐year‐old woman was hospitalized with malnutrition and hypokalemia due to binge eating, self‐induced vomiting, and laxative abuse. Upon detailed history, the loss of control started at an age of approximately 20 years. She started binge eating as a way to de‐stress, and her weight gradually increased to over 70 kg. Subsequently, she developed compensatory behaviors, such as self‐induced vomiting and the usage of laxatives. Since then, her weight had fluctuated between 35 kg and 70 kg, and she had repeated short admissions to the medical ward due to electrolyte disturbances or malnutrition.

When admitted to our ward, she was 158 cm tall and weighed 39.6 kg, with a body mass index (BMI) of 15.9 kg/m^2^. Laboratory tests revealed hypoalbuminemia, renal involvement, liver dysfunction, hyponatremia, and hypokalemia (detailed laboratory data are summarized in Table 1). Abdominal computed tomography revealed multiple hepatic cysts, blunted margins of the liver, irregular convexity of the hepatic surface, and the development of paravalvular veins, all of which indicated cirrhosis. She was diagnosed with the b/p AN that had transitioned from prior BN, according to the *Diagnostic and Statistical Manual of Mental Disorders*, fifth edition.^6^ A few days later, a gradual increase in pleural effusion resulted in acute respiratory failure. Consequently, she required diuretics and artificial ventilation for a week in the ICU. After her physical symptoms improved, psychoeducation was initiated in parallel with nutritional management by nasogastric feeding. She understood BN and b/p AN characteristics clearly and ostensibly had a positive attitude toward tackling BN treatment. Although she tried not to vomit and to stop binge eating, her efforts were temporary. In other words, she could suspend binge eating and self‐induced vomiting for a short period, but her symptoms relapsed upon any stress, especially from mutual non‐understanding with a sibling and with some medical staff. Cognitive behavioral therapy was ineffective for her compensatory behavior. As her physical state improved and her oral intake stabilized, she was discharged 8 months after admission.

**Table 1:** Laboratory data and body weight

	First ad	First dis	Second ad	Second dis	Third ad	Third dis	Fourth ad	Fourth dis	Fifth ad	Fifth dis
BW (kg)	39.6	44.8	51.0		45.2	49.7	47.4	49.7	46.7	56.7
BMI (kg/m^2^)	15.9	18.0	20.4		18.1	19.9	19.0	19.9	18.7	22.7
Alb (g/dL)	3.1	2.9		2.8	3.1	3.1	3.7	2.7	3.8	2.6
BUN (mg/dL)	10.9	10.1		10.1	6.3	22.9	28.4	13.2	25.6	28.9
Cre (mg/dL)	2.29	2.04		2.02	2.25	2.23	3.84	2.23	4.59	2.00
Na (mEq/L)	131	139		140	134	142	130	143	134	140
K (mEq/L)	2.6	3.0		3.1	2.8	4.6	3.2	4.4	3.1	3.7
AST (U/L)	82	25		21	27	30	19	57	29	56
ALT (U/L)	67	16		13	15	44	20	37	51	153
γ‐GT (IU/L)	927	106		75	72	141	137	93	127	134
ALP (U/L)	1984	826		1008	1093	1082	1261	849	1218	772
Hospitalization period	8 months		1 week		2 months		1 week		6 months	
Period between dis and ad	4 months			46 days		5 days		1 week		

Abbreviations: ad, admission; Alb, albumin; ALP, alkaline phosphatase; ALT, alanine transaminase; AST, aspartate transaminase; BUN, blood urea nitrogen; BW, body weight; Cre, creatinine; dis, discharge; GT, γ‐glutamyltransferase; K, potassium; Na, natrium.

After discharge, binge eating, self‐induced vomiting, and laxative abuse persisted. As her compensatory behaviors deteriorated upon stress, she was re‐admitted for another week. However, this short retreat period did not improve her BN symptoms. On the contrary, she overdid her exercise regimen more than before, thereby feeling dizzy and had difficulty walking, which resulted in her readmission 46 days after the end of the short retreat. After hospitalization, laboratory data revealed hypokalemia. Although tube feeding was initiated, neither refeeding syndrome nor edema developed. Gradually, hypokalemia improved, and intake by mouth stabilized. Despite the physical improvement, her urine volume remained low. Therefore, the patient was prescribed diuretics. The patient was discharged approximately 2 months after admission. After discharge, she stayed at home calmly and refrained from exercise; moreover, she was drinking sufficient water. However, she could not stop the self‐induced vomiting or laxative abuse. Consequently, she was admitted due to worsened renal involvement, hypokalemia, and hyponatremia 5 days after discharge. Furthermore, her weight plunged by 2.3 kg and physical examination revealed enophthalmos. In the laboratory data, serum albumin spiked at 3.7 g/dL. These abnormalities indicated dehydration, which possibly worsened renal function. Therefore, the patient was treated with intravenous hydration. One week later, laboratory data and body weight reverted to almost the same status as that during the last discharge. However, she was readmitted 4 days later because of hypokalemia and acute renal failure due to dehydration. Dehydration, renal failure, and hypokalemia were improved by intravenous hydration. Although edema and apnea appeared gradually as her weight reverted, they were improved by switching from spironolactone to furosemide. However, dehydration relapsed whenever she stayed out overnight as she could not stop self‐induced vomiting. Furthermore, she required diuretics because of hypouresis.

Due to vomiting, the nasogastric tube and elemental diet tube came out easily from her stomach and duodenum, respectively. We discussed alternative paths of nutrition and explained the advantages and disadvantages of each method (Table 2). Moreover, the PEG‐J procedure and complications were explained to the patient by a gastroenterologist who later performed the PEG‐J tube placement. As repeated dehydration was expected to worsen her renal function, which might lead to ESRD, we performed gastrostomy insertion with the insertion of a PEG‐J tube. We then attempted to adjust the amount of water and parenteral nutrition (ENORAS) via the PEG‐J tube. Since infusion of ENORAS at 60 mL/h caused reactive hypoglycemia and hyperkalemia, we changed ENORAS to ENEVO, which has less potassium and a lower concentration of carbohydrates. Moreover, we diluted the solution with water and infused it slowly. Furthermore, voglibose was prescribed to prevent hypoglycemia. While self‐induced vomiting and binge eating persisted, dehydration and electrolyte abnormalities did not occur with infusions of 750 mL of ENEVO and 1500 mL of water per day at 100 mL/h during hospitalization.

**Table 2:** Advantages and disadvantages of each nutritional method

Advantages	Disadvantages
*PPN*		
Fewer serious side‐effects Easiest and speediest way of improving dehydration	Cannot provide adequate nutrition; thus, supplemental oral intake is needed Self‐infusion of nutrition is not possible Side‐effects, such as pain, hemorrhage, and nerve injury	
*TPN*		
Enables provision of adequate nutrition Safe and easy infusion when CV port is implanted	Serious side‐effects, such as catheter infections, injury of vital organs, hemorrhage, and pain Self‐infusion of nutrition is not possible Necessity of exchanging catheter periodically or when obstructed incidentally	
*NG tube*		
Natural method of nutrition Enables provision of adequate nutrition Self‐infusion of nutrition is possible	May be decannulated incidentally or intentionally, especially by vomiting Possibility of gastrointestinal perforation Skin problems Necessity of exchanging tubes periodically or when obstructed incidentally Cosmetically distressing Continuous discomfort	
*PEG‐J tube*		
More natural nutrition than intravenous nutrition Enables provision of adequate nutrition Self‐infusion of nutrition is possible Low incidence of decannulation by vomiting Less frequency of exchanging tubes than NG tube The tube is usually invisible	May be decannulated incidentally or intentionally Possibility of gastrointestinal perforation Skin problems Infection in the placement area Pain and itch Necessity of exchanging tubes periodically or when obstructed incidentally	

Abbreviations: CV port, central venous port; NG tube, nasogastric tube; PPN peripheral parenteral nutrition; TPN, total parenteral nutrition.

She was discharged approximately 6 months after admission. Although her sibling strictly managed laxatives after discharge, her occult laxative abuse caused frequent diarrhea. Thus, slight dehydration and hypokalemia sometimes occurred, and ENEVO and water were increased up to 1000 mL and 2000 mL, respectively. An increase in the infusion rate of this mixed fluid to 450 mL/h did not cause hypoglycemia. However, hypoglycemia occurred when she was once infused with the fluid at 600 mL/h. Even when dehydration and hypokalemia occurred, she was able to revert quickly by intravenous hydration in our outpatient room.

Stable nutrition via PEG‐J prevented dehydration. A weekly check of her blood biochemistry revealed no worsening of renal function. Her stable physical state allowed us to provide interpersonal therapy. Vomiting, binge‐eating, and unsafe compensatory behaviors decreased as she improved her interpersonal skills.

## DISCUSSION

This case suggested two important findings. First, chronic BN or b/p AN patients may tend to repeat dehydration, which may lead to ESRD. Second, repeated dehydration may be prevented by hydration via a PEG‐J tube.

The repeated dehydration tendency can be explained by reduced colloid osmotic pressure, laxatives adding to diuretic use, and impaired gastric emptying. Although serum albumin level of AN/BN patients is usually within the normal range, some cases show hypoalbuminemia.^7^ A previous report suggests that hypoalbuminemia with BN may be caused by occult infection.^8^ In our case, although we did not suspect occult infection, hypoalbuminemia may have been caused by not only malnutrition, but by albumin‐synthetic depression upon liver dysfunction. Liver dysfunction in AN/BN patients is explained by hepatocyte autophagy, depletion of glutathione, hypoperfusion, and ischemia.^3^ Despite normalization of pre‐albumin levels, hypoalbuminemia slightly improved. This indicates that chronic liver dysfunction had already progressed in cirrhosis, and that her liver was not able to synthesize albumin sufficiently. Reduced colloid osmotic pressure due to hypoalbuminemia tends to shift the free water to the third space. Consequently, hypoalbuminemia leads to volume depletion.

On the other hand, renal involvement was caused by repeated hypokalemia and hydration as much as overfiltration by binge‐eating.^9^ Volume depletion and renal involvement led to urine decrease and edema. Therefore, diuretics are continuously required. Although strict management of nutrition and medications ostensibly stabilized her water balance, she was dehydrated due to laxative abuse after discharge. As a result, diuretic effects, vomiting, and laxative abuse led to further hypokalemia. To make matters worse, hypokalemia caused hypoperistalsis, which led to delayed gastric emptying.^1^ This means that the loss of fluid and electrolytes increased upon vomiting, and dehydration worsened it further. As discussed thus far, liver dysfunction, renal dysfunction, diuretics, and laxatives cause a vicious cycle of dehydration in BN and b/p AN patients. Hence, we performed percutaneous endoscopic gastrostomy and placed a PEG‐J tube. After placement, dehydration was prevented even though she continued with abusing diuretics, binge eating, and self‐induced vomiting. A few previous reports had conducted percutaneous endoscopic gastrostomy,^10^ but to the best of our knowledge, no previous study has reported that PEG‐J prevents ESRD in chronic BN patients.

Our case report has two major limitations. First, there was a lack of long‐term prognosis after PEG‐J tube placement. Although hypoglycemia had already been encountered, other medical problems, such as infection, may occur in the future. In particular, the site of insertion of PEG‐J is susceptible to infections. Once infected, it may be difficult to heal due to malnutrition.^10^ Therefore, chronic wounds may cause sepsis, leading to death. Second, the findings of the present case cannot be generalized to patients with contraindications to PEG and PEG‐J. For example, hepatomegaly, splenomegaly, and/or gastric varices are relative contraindications, and the existence of severe ascites and interposed organs are absolute contraindications.^5^ Since such contraindications are relatively common in patients with chronic BN, careful attention is required when considering PEG‐J.

## CONCLUSION

Chronic BN or b/p AN lead to dehydration, which finally may cause ESRD. To prevent this, the placement of a PEG‐J tube and intake fluid via PEG‐J may be useful.

## AUTHOR CONTRIBUTIONS

Kazuhito Takahashi and Takaaki Shimizu conceived the idea of study conceptualization. Kazuhito Takahashi wrote the draft of the manuscript. Daimei Sasayama and Shinsuke Washizuka supervised and contributed to the writing of the manuscript. All authors approved the final version of the manuscript to be published.

## CONFLICT OF INTEREST

The authors declare no conflict of interest.

## ETHICS APPROVAL STATEMENT

We obtained written informed consent from the patient for publication of this case report.

## PATIENT CONSENT STATEMENT

We obtained written informed consent from the patient for publication of this case report.

## CLINICAL TRIAL REGISTRATION

N/A.

## Data Availability

N/A.
